# Diagnosis, investigation and management of hereditary spastic paraplegias in the era of next-generation sequencing

**DOI:** 10.1007/s00415-014-7598-y

**Published:** 2014-12-06

**Authors:** Anke Hensiek, Stephen Kirker, Evan Reid

**Affiliations:** 1 Department of Neurology, Cambridge University Hospitals NHS Trust, Addenbrooke’s Biomedical Campus, Cambridge, UK; 2 Addenbrooke’s Rehabilitation Clinic, Cambridge University Hospitals NHS Trust, Addenbrooke’s Biomedical Campus, Cambridge, UK; 3 Cambridge Institute for Medical Research, University of Cambridge, Addenbrooke’s Biomedical Campus, Cambridge, CB2 0XY UK; 4 Department of Medical Genetics, University of Cambridge, Addenbrooke’s Biomedical Campus, Cambridge, UK

**Keywords:** Hereditary spastic paraplegia, Axonal degeneration, Pure HSP, Complex HSP with cerebellar ataxia, Thin corpus callosum

## Abstract

The hereditary spastic paraplegias (HSPs) are a group of genetic conditions in which spastic paralysis of the legs is the principal clinical feature. This is caused by a relatively selective distal axonal degeneration involving the longest axons of the corticospinal tracts. Consequently, these conditions provide an opportunity to identify genes, proteins and cellular pathways that are critical for axonal health. In this review, we will provide a brief overview of the classification, clinical features and genetics of HSP, highlighting selected HSP subtypes (i.e. those associated with thin corpus callosum or cerebellar ataxia) that are of particular clinical interest. We will then discuss appropriate investigation strategies for HSPs, suggesting how these might evolve with the introduction of next-generation sequencing technology. Finally, we will discuss the management of HSP, an area somewhat neglected by HSP research.

## Introduction

The hereditary spastic paraplegias (HSPs) are a group of genetic conditions in which spastic paralysis of the legs is the principal clinical feature [1, 2]. In most subtypes of HSP, this is caused by a relatively selective distal axonal degeneration involving the longest axons of the corticospinal tracts [3]. As a group, these conditions are moderately rare, with prevalence estimates in different geographical regions ranging from 0.1 to 9.6 per 10^5^ [4]. However, as they are chronic and sometimes severe conditions that frequently present in childhood and young adult life, they represent a significant disease burden. In addition, they are scientifically important, since they provide an opportunity to identify genes, proteins and cellular pathways that are critical for axonal health.

In this review, we will provide a brief overview of the classification, clinical features and genetics of HSP, highlighting selected HSP subtypes that are of particular clinical interest. We will then discuss appropriate investigation strategies for HSPs, suggesting how these might evolve with the introduction of next-generation sequencing technology. Finally, we will discuss the management of HSP, an area somewhat neglected by HSP research.

## Overview of main HSP clinical categories

Traditionally, the hereditary spastic paraplegias are subcategorised into “pure” and “complex” (or “complicated”) subtypes [3]. In northern European populations, pure forms are the most frequent. The clinical features of pure HSP have been thoroughly described in previous research articles and reviews, and diagnostic criteria have been proposed [1, 5–9]. Briefly, the typical clinical picture is of a slowly progressive, predominantly symmetrical, spastic paraplegia. This is frequently accompanied by minor sensory abnormalities (such as absent vibration sensation) and neurological bladder involvement, but bowel involvement is rare. If seen at all, arm involvement is minimal and does not extend beyond hyper-reflexia and minor weakness (e.g. difficulty unscrewing a tight bottle top); more significant arm involvement should raise the possibility of alternative diagnoses such as primary lateral sclerosis or amyotrophic lateral sclerosis, while bulbar involvement is incompatible with a diagnosis of pure HSP. Age at onset varies from childhood to late adult life. Within many multiplex families it is highly variable, although mutations in certain genes, notably spastic paraplegia gene (SPG) 3A/atlastin1, are predominantly associated with a childhood age at onset.

While pure HSPs present a relatively homogenous clinical picture, complex HSPs are a disparate group of distinct disorders (Table 1) [1–3]. As most complex HSPs are inherited in an autosomal recessive pattern, they are the commonest forms of HSP in populations where consanguinity is frequent; for example, in a Tunisian series of 38 families, approximately 70 % of families had autosomal recessive complex HSP [10]. However, it is important to recognise that these complex subtypes of HSP are found worldwide, e.g. HSP with thin corpus callosum caused by SPG11 or SPG15 mutations has been described in many populations and is the most common type of complex HSP that we see in our own clinical practice in Cambridge, UK.

**Table 1:** List of known or probable HSP genes

Gene	Protein	Inherited	OMIM	Comment	P/C*	Clinical features of complex forms
SPG1/L1CAM	L1-cell adhesion molecule	XL	#303350		C	1. MASA syndrome, 2. X-linked hydrocephalus, 3. X-linked complicated HSP
SPG2/PLP1	Proteolipid protein 1	XL	#312920		P/C	1. Pure HSP, 2. complex HSP, 3. Pelizaeus–Merzbacher disease
SPG3A/ATL1	Atlastin1	AD	#182600		P	
SPG4/SPAST	Spastin	AD	#182601		P	
SPG5A/CYP7B1	25-hydroxycholesterol 7-alpha-hydroxylase	AR	#270800		P	
SPG6/NIPA1	NIPA1	AD	#600363		P	
SPG7	Paraplegin	AR	#607259		P/C	Cerebellar ataxia, optic atrophy, deafness, amyotrophy
SPG8/KIAA0196	Strumpellin	AD	#603563		P	
SPG9	–	AD	%601162		C	Cataracts, motor neuropathy, skeletal abnormalities and gastro-oesophageal reflux.
SPG10/KIF5A	Kinesin heavy chain 5A	AD	#604187		P/C	Peripheral neuropathy, amyotrophy, mental retardation, parkinsonism
SPG11	Spatacsin	AR	#604360		C	HSP with thin corpus callosum. Peripheral neuropathy, intellectual disability, cognitive decline, amyotrophy, pseudobulbar involvement, cerebellar involvement, parkinsonism, dystonia
SPG12/RTN2	Reticulon2	AD	#604805		P	
SPG13/HSPD1	Heat shock 60 kDa protein 1	AD	#605280		P	
SPG14	–	AR	%605229		C	Intellectual disability, motor neuropathy
SPG15	Spastizin	AR	#270700		C	HSP with thin corpus callosum. Peripheral neuropathy, intellectual disability, cognitive decline, amyotrophy, pseudobulbar involvement, cerebellar involvement, parkinsonism, dystonia, pigmentary maculopathy (Kjellin syndrome).
SPG16	–	XLR	%300266		C	Spastic quadraplegia, intellectual disability, cerebellar ataxia, optic atrophy, nystagmus, bowel and bladder dysfunction.
SPG17/BSCL2	Seipin	AD	#270685		C	Silver syndrome; HSP with distal amyotrophy. Overlaps with HMSN V.
SPG18/ERLIN2	ER lipid raft associated 2	AR	#611225		C	Epilepsy, intellectual disability, pseudobulbar palsy, joint contractures
SPG19		AD	%607152		P	
SPG20/SPARTIN	Spartin	AR	#275900		C	Troyer syndrome: dysarthria, pseudobulbar palsy, intellectual disability, amyotrophy, short stature
SPG21/MAST	Maspradin	AR	#248900		C	Mast syndrome: progressive dementia, cerebellar signs, extra-pyramidal involvement, thin corpus callosum.
SPG22	SLC16A2	XLR	#300523		C	Allan-Herndon-Dudley syndrome
SPG23	–	AR	%270750		C	Disordered skin pigmentation, peripheral neuropathy
SPG24	–	AR	%607584		P	
SPG25	–	AR	%608220		C	Disc herniation
SPG26/B4GALNT1	Beta-1,4 *N*-acetylgalactosaminyltransferase 1	AR	#609195		C	Intellectual disability, peripheral neuropathy, dysarthria, cerebellar signs, extrapyramidal involvement, cortical atrophy
SPG27	–	AR	%609041		P	
SPG28/DDHD1	Phospholipase DDHD1	AR	#609340		P/C	Cerebellar eye signs, peripheral neuropathy
SPG29/KIF1A	Kinesin family member 1A	AD	%609727			Sensorineural hearing impairment, neonatal hyperbilirubinemia, hiatus hernia
SPG30	–	AR	#610357		C	Cerebellar signs, peripheral neuropathy
SPG31/REEP1	Receptor expression-enhancing protein 1	AD	#610250		P	
SPG32	–	AR	%611252		C	Cognitive impairment, thin corpus callosum, cortical atrophy, cerebellar atrophy, pontine dysraphia.
[SPG33/ZFYVE27]	[Protrudin]	[AD]	#610244	Mutation may not be pathogenic-present in control populations	[P]	
SPG34	–	XLR	%300750		P	
SPG35/FA2H	Fatty acid 2-hydroxylase	AR	#612319		C	Dysarthria, intellectual decline, leukodystrophy, dystonia, optic atrophy, seizures, cerebellar signs, thin corpus callosum. May cause brain iron accumulation.
SPG36	–	AD	%613096		C	Lower limb sensory changes
SPG37	–	AD	%611945		P	
SPG38	–	AD	%612335		C	Similar to Silver syndrome (see SPG17)
SPG39/PNPLA6	Neuropathy target esterase	AR	#612020		C	Distal amyotrophy, cerebellar atrophy. Allelic with Boucher–Neuhauser syndrome (spinocerebellar ataxia, hypogonadotropic hypogonadism, chorioretinal dystrophy)
SPG40	–					
SPG41	–	AD	%613364	Single family with lod score <3		
SPG42/SLC33A1	Acetyl-coenzyme A transporter 1	AD	#612539		P	
SPG43/C19orf12	C19orf12	AR	#615043		P/C	Upper limb involvement, distal amyotrophy. May also be associated with brain iron accumulation
SPG44/GJC2	Gap junction gamma-2 protein	AR	#613206		C	Cerebellar signs, seizures, cognitive impairment, scoliosis, leukodystrophy, thin corpus callosum.
SPG45/NT5C2	Cytosolic purine 5’-nucleotidase	AR	#613162		C	Optic atrophy, thin corpus callosum, intellectual disability
SPG46/GBA2	Non-lysosomal glucosylceramidase	AR	#614409		C	Cerebellar signs, intellectual impairment, cerebral atrophy, cerebellar atrophy, thin corpus callosum, pseudobulbar involvement, cataracts.
SPG47/AP4B1	AP-4 complex subunit beta-1	AR	#614066		C	Neonatal hypotonia, severe intellectual impairment, dysmorphic features, thin corpus callosum.
SPG48/AP5Z1	AP-5 complex subunit zeta-1	AR	#613647		C	Urinary incontinence
SPG49/TECPR2	Tectonin beta-propeller repeat-containing protein 2	AR	#615031		C	Intellectual impairment, dysmorphic features, cerebral atrophy, cerebellar atrophy, thin corpus callosum
SPG50/AP4M1	AP-4 complex subunit mu-1	AR	#612936		C	Neonatal hypotonia, severe intellectual impairment, pseudobulbar signs, microcephaly, cerebellar atrophy
SPG51/AP4E1	AP-4 complex subunit epsilon-1	AR	#613744		C	Neonatal hypotonia, severe intellectual impairment,dysmorphic features, seizures, cortical atrophy, cerebellar atrophy, microcephaly
SPG52/AP4S1	AP-4 complex subunit sigma-1	AR	#614067		C	Neonatal hypotonia, severe intellectual impairment, microcephaly, dysmorphic features, short stature
SPG53/VPS37A	Vacuolar protein sorting-associated protein 37A	AR	#614898		C	Intellectual disability
SPG54/DDHD2	Phospholipase DDHD2	AR	#615033		C	Intellectual disability, dysarthria, dysphagia, optic hypoplasia, thin corpus callosum and white matter changes, short stature
SPG55/C12ORF65	C12orf65	AR	#615035		C	Peripheral neuropathy, optic atrophy, intellectual disability
SPG56/CYP2U1	Cytochrome P450 2U1	AR	#615030		P/C	Upper limb involvement, peripheral neuropathy, intellectual impairment, thin corpus callosum
SPG57/TFG	Protein TFG	AR	#615658		C	Optic atrophy, peripheral neuropathy
SPG58/SPAX2/KIF1C	Kinesin-like protein KIF1C	AR	#611302		C	Cerebellar ataxia
SPG59/USP8	Ubiquitin carboxyl-terminal hydrolase 8	AR	*603158	VUS	P	
SPG60/WDR48	WD repeat-containing protein 48	AR	*612167	VUS	C	Nystagmus, peripheral neuropathy, intellectual disability
SPG61/ARL6IP1	ADP-ribosylation factor-like protein 6-interacting protein 1	AR	#615685		C	Peripheral neuropathy
SPG62	–					
SPG63/AMPD2	AMP deaminase 2	AR	#615686		P/C	White matter changes in corpus callosum
SPG64/ENTPD1	Ectonucleoside triphosphate diphosphohydrolase 1	AR	#615683		C	Dysarthria, intellectual disability, microcephaly, delayed puberty
SPG65	Duplicate of SPG45					
SPG66/ARSI	Arylsulfatase I	AR	*610009	VUS	C	Thin corpus callosum, cerebellar hypoplasia, colpocephaly, peripheral neuropathy
SPG67/PGAP1	GPI inositol-deacylase	AR	*611655	VUS	C	Intellectual disability, tremor, absent corpus callosum, defective myelination
SPG68/FLRT1	leucine-rich repeat transmembrane protein FLRT1	AR	*604806	VUS	C	Optic atrophy, peripheral neuropathy
SPG69/WARBM2/RAB3GAP2	Rab3 GTPase-activating protein non-catalytic subunit	AR	*609275		C	Warburg micro syndrome, Martsolf syndrome
SPG70/MARS	Methionine-tRNA ligase, cytoplasmic	AR	*156560	VUS	P/C	Mild intellectual disability
SPG71/ZFR	Zinc finger RNA-binding protein	AR	*615635	VUS	C	Thin corpus callosum
SPG72/REEP2	Receptor expression-enhancing protein 2	AR/AD	#615625		P	
Spastic ataxias						
SPAX1/VAMP1	Vesicle-associated membrane protein 1	AD	#108600		SPAX	
SPAX2/SPG58/KIF1C	Kinesin-like protein KIF1C	AR	#611302		SPAX	
SPAX3/MARS2	Methionine-tRNA ligase, mitochondrial	AR	#611390		SPAX	
SPAX4/MTPAP	Poly(A) RNA polymerase, mitochondrial	AR	#613672		SPAX	
SPAX5/AFG3L2	AFG3-like protein 2	AR	#614487		SPAX	
SPAX6/SACS	Sacsin	AR	#270550		SPAX	Spastic ataxia of Charlevoix-Saguenay
EXOSC3/PCH1B	Exosome complex component RRP40	AR	*606489	Allelic with pontocerebellar hypoplasia		

Note that sometimes the distinction between pure and complex HSP may be somewhat arbitrary. See OMIM entries (http://www.ncbi.nlm.nih.gov/omim/) for the appropriate primary literature

*AD* autosomal dominant, *AR* autosomal recessive, *C* complex, *HMSN* hereditary motor and sensory neuropathy, *P* pure, *SPAX* spastic ataxia, *VUS* variant of uncertain significance

## Genetics of HSPs

The HSPs show a remarkable degree of genetic heterogeneity. The chromosomal location of more than 70 SPGs is known, and presently more than 50 of the genes have been fully identified (Table 1). Moreover, additional syndromes such as Warburg Micro syndrome, previously considered distinct, have recently been incorporated under the rubric of HSP by reason of overlapping cellular pathology [11]. This new knowledge has led to an evolution of the classification of HSP, which, while still referring to phenotypic information, is increasingly focussed on the gene involved. In addition, gene identification has resulted in the distinction between pure and complex HSP becoming somewhat blurred as it has become apparent that mutations in the same gene may cause either a pure or complex phenotype. An example of this is the rare identification of mutations in SPG4/SPAST, SPG31/REEP1 or SPG3A/atlastin1, three genes typically associated with pure HSP, in patients who have spastic paraplegia accompanied by cerebellar ataxia or peripheral neuropathy [12–14].

### Pure HSP phenotype

In northern European populations, autosomal dominant inheritance accounts for the majority of patients from families with pure HSP [15]. Autosomal recessive inheritance accounts for most of the remainder, while clear-cut X-linked inheritance is infrequent. Rarely, gonadal mosaicism for a dominant gene has been described and this can be mistaken for autosomal recessive inheritance [16]. In addition, some patients present without a family history. Potential genetic explanations for this are varied and include singleton autosomal recessive cases, new autosomal dominant mutations, or inherited autosomal dominant mutations with non-penetrance in a parent. The estimated relative frequency of mutations in selected genes associated with pure HSP is given in Table 2. From this table, it can be seen that testing for three genes, SPG4/SPAST, SPG3A/Atlastin1 and SPG31/REEP1, will identify the responsible mutation in approximately 50 % of families with autosomal dominant pure HSP. For SPG4/SPAST, this testing should include methodologies to pick up whole exon deletions, as these are a common mutational class in this subtype of HSP (they have also been described in other forms including SPG3A/Atlastin1 and SPG31/REEP1-HSP) [14, 17–19]. For families with an autosomal recessive inheritance pattern, screening SPG5 (CYP7B1) will pick up the causative mutations in approximately 5–10 % of cases. However, the yield from testing each individual additional gene beyond these commoner genes is low. Using traditional “one at a time” sequential gene testing to detect these rare mutations can be very expensive and may not be justifiable for publically funded health services. Next-generation sequencing approaches are currently revolutionising this situation (see below).

**Table 2:** Reported frequencies of mutations in selected pure HSP genes

Gene	Reported frequency in familial cases	Frequency in sporadic cases	Frequency in unselected cases
SPG4/SPAST	31–47 % of AD-pure HSP [49–54]	7–18 % [51, 52, 55–57]	17–26 % [51, 56, 58]
SPG3A/ATLASTIN1	8–39 % of AD-pure HSP (studies often comprised families with early onset and screened SPG4 negative) [51, 59–61]	Unknown	7 % (after exclusion of SPG4 mutations) [13]
SPG31/REEP1	2–8 % of AD-pure HSP (screened families typically SPG4 and/or SPG3 negative) [62–64]	2 % [65]	3–7 % [63, 65]
SPG10/KIF5A	3–5 % (series included families with complicated HSP, typically SPG4 and SPG3 negative). A rare cause of pure HSP [66–68]	None detected	Unknown
SPG8/KIAA0196	5–8 % of SPG3A and SPG4-negative AD HSP families [69, 70]	Unknown	Unknown
SPG12/RTN2	Rare [71]	Rare	Rare
SPG6/NIPA1	<1 % of AD-pure HSP [72, 73]	Unknown	Rare [72]
SPG13/HSPD1	Rare	Unknown	Unknown
SPG42/SLC33A1	Unknown (single family only)	Unknown	Unknown
SPG5A/CYP7B1	7 % of AR-pure HSP 1 reported AD family [30]	3 % [30]	Unknown
SPG7	Rare cause of AR-pure HSP	7–12 % [34, 74]	<5 % of unselected AR families [75, 76]

### Complex HSP phenotypes

As mentioned above, the complex HSPs are a diverse set of conditions, each with distinctive clinical features and each caused by one or a small set of genes. Here, we will focus on two of the more common presentations, HSP with thin corpus callosum and HSP accompanied by cerebellar ataxia.

#### HSP with thin corpus callosum

A thin corpus callosum on MRI scanning is a characteristic feature of patients who have autosomal recessive SPG11 and SPG15 mutations. These patients have a variable combination of clinical features which, as well as thin corpus callosum, includes typically childhood or teenage onset spastic paraplegia, learning difficulties with progressive cognitive decline, cerebellar signs, ocular involvement, peripheral motor axonopathy and extrapyramidal features including parkinsonism. SPG11/15 mutations are associated with relatively severe gait involvement and patients often eventually require a wheelchair. It is important to note that a thin corpus callosum is not a universal finding in SPG11/15 HSP (e.g. in large series, it was found in 90 % of SPG11 cases and 60 % of SPG15 patients), and that learning difficulties may be present before the spastic gait develops [20–22].

Cell biological studies on the SPG11 and SPG15 proteins have revealed why mutations in these genes lead to a similar clinical phenotype. Both SPG11 and SPG15 proteins interact with a 4-protein complex termed AP5 (adapter protein 5), which localises to small intracellular membrane-bound vesicles called endosomes [23, 24]. Although AP5’s function is not fully understood, it probably acts to sort membrane cargoes away from the endosomal compartment [23, 24]. Intriguingly, recessive mutations in a component of the core AP5 complex (SPG48/AP5Z1) also cause a very similar clinical phenotype to SPG11/15 mutations, with cognitive defects, thin corpus callosum and spastic paraplegia [25]. Thus, HSP with thin corpus callosum is an excellent example of the principle that diseases with similar phenotypes are often caused by mutations in functionally related genes. At least four other subtypes of recessive HSP may also present with thin corpus callosum, SPG21/Maspardin, SPG35/FA2H and SPG46/GBA2 and SPG54/DDHD2 ([25] and see Table 1); it remains to be seen whether any of the encoded proteins are functionally related to the AP5 complex.

#### HSP with cerebellar ataxia

Patients who present with a mixed cerebellar ataxia and spastic paraplegia phenotype represent a particular diagnostic challenge, as the differential diagnosis is very broad. It can be helpful to first make a decision as to whether spastic paraplegia or cerebellar ataxia is the dominant feature. There are consensus diagnostic pathways for patients with predominant cerebellar ataxia and we will not review these further here [26]. In patients where spastic paraplegia is the predominant feature, it is useful to subdivide into pure and complex subtypes, analogous to the subdivision of HSP.

*“Complex” spastic ataxia* in which the cerebellar ataxia is a (sometimes variable) feature of a more complex syndromal picture. Such conditions include HSP with thin corpus callosum (SPG11/15 mutations), SPG35/FA2H-associated HSP, Troyer syndrome (SPG20) and Mast syndrome (SPG21), SPG26/B4GALNT1, SPG30/KIF1A, SPG39/PNPLA6 and SPG46/GBA2. This group also contains a variety of rare metabolic conditions (reviewed in [27, 28]), including cerebrotendinous xanthomatosis, triple H syndrome, cerebral folate deficiency, metachromatic leukodystrophy, Type III 3-methylglutaconic aciduria, as well as other conditions such as Alexander disease and vanishing white matter disease. Rare patients with Chediak–Higashi syndrome (caused by mutations in the lysosomal trafficking regulator LYST gene) may also present with spastic paraplegia, cerebellar ataxia and peripheral neuropathy, without the hypopigmentary or immune deficiency typically associated with this condition [29].

*“Pure” spastic ataxia* There are many patients in whom spastic paraplegia with cerebellar involvement is the exclusive clinical presentation. This can occur in three main scenarios:

- As an unusual presentation of mutations in genes that are classically associated with “pure” hereditary spastic paraplegia, including autosomal dominant SPG4/spastin and SPG31/REEP1 and autosomal recessive SPG5/CYP7B1 [12, 14, 30].
- As a presentation of genes that are classically associated with cerebellar ataxia. This would especially include Friedreich ataxia and SCA3, in which pyramidal signs may be the presenting feature [31, 32].
- As a presentation of mutations in genes that typically cause a spastic paraplegia/cerebellar ataxia overlap syndrome. These genes are often classified under the spastic ataxia (SPAX) nomenclature. Well-recognised examples include the autosomal recessive spastic ataxia of Charlevoix-Saguenay (ARSACS; SPAX6) and SPG7/paraplegin [33, 34]. They also include VAMP1 (SPAX1), KIF1C gene (SPAX2/SPG58), MARS2 (SPAX3), MTPAP (SPAX4) and AFG3L2 (SPAX5/SCA28) (Table 1) [32, 35].

## Investigation of a patient with suspected HSP

The traditional approach to investigating a patient with possible HSP is to define the phenotype and inheritance pattern, exclude alternative causes, then attempt to make a definitive molecular genetic diagnosis, where possible using clinical and family history information to focus genetic investigations appropriately.

### Definition of phenotype, inheritance and exclusion of other causes

As specific investigations of a patient with suspected HSP depend on their clinical features and inheritance pattern, careful clinical phenotyping of index cases and potentially affected family members is crucial. A detailed medical history should include developmental milestones and a three generational family tree. Affected individuals and ideally, apparently unaffected family members should undergo a clinical neurological examination, to document specific neurological features and identify asymptomatic family members who may have subtle abnormal signs on examination. This is particularly important for apparently sporadic cases or isolated sibships, where positive examination findings in an asymptomatic parent will point towards the probability of autosomal dominant inheritance.

Even in individuals who have typical clinical features of HSP and a positive family history of spastic paraparesis, it is important to consider alternative causes and importantly exclude other treatable familial conditions. In his regard, MR imaging of the brain and spinal cord is crucial and should be undertaken in all sporadic cases and at least one family member of familial presentations. Imaging will not only identify many structural, inflammatory or metabolic abnormalities but is also useful to guide investigations into specific genetic causes (for example, by highlighting atrophy patterns). Note that imaging does have an appreciable false negative rate, for example, it can be normal in certain metabolic or inflammatory conditions, including adrenoleukodystrophy or primary progressive multiple sclerosis.

A baseline metabolic blood screen in the index case could include Vitamins B12 and E, creatine kinase, very long chain fatty acids, white cell enzymes, anti-nuclear antibody and copper levels. However, individuals with more complex clinical features or an unclear family history may require further baseline tests. For example, in presumed sporadic pure HSP, a set of more extensive investigations is appropriate, as an inherited basis for the disease has not been proven. This includes CSF examination (including oligoclonal bands, HTLV serology), Syphilis and HIV serology as well as vasculitis screen [36–38]. An oral short trial of Levodopa will identify those individuals with dopa-responsive dystonia and neurophysiology can be useful if there is an associated neuropathy or suspicion of amyotrophic lateral sclerosis [39]. Table 3 summarises a list of conditions to be considered in the differential diagnosis and investigation of sporadic pure HSP.

**Table 3:** Conditions and investigations to be considered in the differential diagnosis of pure HSP

Structural and vascular	
Arterio-venous dural fistula [77]	MRI/angiogram
Spinal or parasagittal tumour	MRI
Spondylosis	MRI
Inflammatory	
Multiple Sclerosis	MRI, CSF
Vasculitic Myelopathy [36]	Autoimmune profile
Stiff person syndrome [78]	Neurophysiology, antibody testing
Sarcoidosis [79]	MRI, CSF, chest X-ray
Metabolic (acquired and hereditary)	
Vitamin deficiency (B12, E)	Vitamin levels
Nitrous oxide toxicity [80]	History, B12 level
Adrenoleucodystrophy and other leucodystrophies [81, 82]	White cell enzymes, VLCFA, MRI
Copper deficiency myelopathy [83]	Copper levels
Degenerative	
Primary lateral sclerosis	Neurophysiology and evolution of clinical picture
Infectious	
Tropical spastic paraparesis [37]	HTLV—1 serology, CSF
HIV myelopathy [38]	HIV serology, CSF
Syphilis	Syphilis serology, CSF
Other	
Radiation myelopathy [84]	History, imaging
Spinocerebellar ataxias and other genetic conditions (see text and [39])	Genetic testing. Trial of l-dopa

For those individuals in whom the above baseline investigations are unremarkable and/or where the family history and clinical features point towards a specific genetic diagnosis, it is then appropriate to test for the suspected mutation.

### Genetic investigations

There is considerable clinical utility for HSP families in detecting their causative mutation. It gives diagnostic certainty, prevents further unnecessary investigations and opens the possibility of predictive and prenatal testing. However, until very recently, in many healthcare systems, economic factors necessitated that genetic investigations were restricted to analysis of HSP genes in which there was the highest chance of detecting a mutation, as the expense of analysing rarely-causative genes was not justified by the low mutation detection rate per additional gene analysed. However, this has changed with the introduction of next-generation sequencing, which allows cost-effective analysis of many genes together, using approaches including (1) sequencing of large panels of genes, (2) sequencing of all coding exons in the genome, or (3) sequencing of entire genomes. At present, a common approach uses next-generation sequencing to screen exons of panels of genes causally associated with specific conditions or groups of conditions. These new approaches hold the prospect of substantially increasing the mutational pick-up rate for HSP patients. Once fully integrated into molecular diagnostic services, it is possible to envisage that genetic testing could be introduced at an early stage in patient work-up, perhaps avoiding the need for other costly or painful investigations if a definitive molecular genetic diagnosis is made.

Although these developments are undeniably positive, there are pitfalls and caveats associated with next-generation sequencing approaches. Sequence variation is very common in the genome (for example, many millions of single nucleotide variants have been identified) [40], with the vast majority of these sequence changes having a neutral effect with no clinical consequence. In addition, many of these sequence changes are individually rare. Thus, next-generation approaches typically detect numerous rare sequence variants; the more genes that are analysed, the larger this problem becomes. A major problem lies in deciding which, if any, of the detected sequence changes are pathogenic. In most cases, this can be resolved using bioinformatics and other approaches (e.g. identifying previously reported and validated mutations, excluding known rare population polymorphisms, determining the likely effect on the amino acid sequence of the encoded protein and how deleterious this effect is likely to be, excluding mutations that do not co-segregate with disease) [41]. However, inevitably in some cases, a single conclusively pathogenic mutation cannot be identified and one or more candidate pathogenic mutations remain. This is a particular issue with mutations of the missense class (in which only a single amino acid of the encoded protein is altered), since it can be difficult to predict whether the resulting effect on the protein will have functional consequences. Such sequence changes are often reported as variants of uncertain significance. Defining approaches to determining which of these mutations are pathogenic will be a major future challenge, as will be determining ethical approaches to whether and how the uncertainty associated with such sequence changes is reported back to patients.

In addition, exon-sequencing approaches do not detect all mutational classes. A particular problem arises in detecting large-scale deletions or duplications involving whole exons, or in detecting promoter or deep intronic mutations. This is an issue for at least some HSP genes, e.g. whole exon deletions are a common cause of SPG4/SPAST HSP (see above). We anticipate that in the relatively near future this issue will be resolved by whole-genome sequencing, which has the capacity to identify large deletions/duplications, as well as promoter and deep intronic mutations. In a research setting, genome sequencing may also allow identification of causative mutations in genes that have not previously been associated with the disease under consideration. In the meantime, additional testing for deletions/duplications should be considered in selected genes for appropriate patients, prioritised based on clinical features, in whom mutations have not been identified in exon-sequencing panels.

High-throughput sequencing approaches also may present ethical issues if so-called “incidental findings” (clinically relevant changes in genes unrelated to the condition being tested) are detected. These are particularly important when whole-exome or whole-genome approaches are taken, where, given sufficient numbers of tested subjects, it is inevitable that other potentially clinically significant abnormalities, e.g. mutations in cancer predisposing genes or detection of carrier status for autosomal recessive disease, will be detected. Whether and how such findings are reported back to patients requires careful consideration of their clinical validity and utility, and the ethical issues surrounding this are currently a topic of much debate [42].

Finally, while next-generation sequencing approaches have the potential to increase rates of molecular genetic diagnosis in HSP, they do not remove the need for careful phenotyping––this is still important, as it can help to focus bioinformatics analysis onto the most relevant genes. Careful phenotyping may also better define the clinical spectrum associated with pathogenic mutations in particular genes.

## Rehabilitation and therapy for HSP patients

People with HSP complain of muscle stiffness, pain, spasms and cramps, tripping over their toes due to weakness of ankle dorsiflexion and hip flexion, loss of balance, effortful walking and progressively more flexed standing posture. Eventually, walking becomes impossible for some patients due to a combination of (a) spasticity, (b) weakness, particularly of ankle dorsiflexion, leading to (c) loss of range of movement at ankle, knee and hip, making it impossible to stand straight, and (d) loss of motor control leading to delayed postural reflexes and loss of balance.

A home exercise programme supervised by a physiotherapist, concentrating on stretches to maintain range of movement and reduce spasticity, accompanied by balance exercises in patients with more advanced disease, is the cornerstone of management. This is usually supported by a trial of oral muscle relaxants such as Baclofen, Tizanidine, or Gabapentin/Pregabalin. Problematic spasticity in specific groups of muscles, most commonly in the ankle plantarflexors and hip adductors, may be treated by Botulinum toxin without the risk of sedation associated with oral muscle relaxants [43]. The role of Botulinum toxin is to support and facilitate stretching and splinting, rather than to replace it. Functional electrical stimulation (FES) is popular among patients and physiotherapists and is as effective as simple off the shelf ankle foot orthoses (AFOs) in the early stages before calf shortening has developed: after that, FES is less useful as it cannot provide support or compensation during stance phase [44–46].

Intrathecal Baclofen, delivered by an implanted pump, is the most effective method of reducing very high tone in the lower limbs, and can bring immediate relief of pain and improvement in sitting posture, along with a reduction in effort transferring from wheelchair to bed or car [47]. Effective control of muscle tone often improves quality of sleep for the patient and their partner, and permits stretching and splinting with the aim of preventing further deterioration of flexion contractors. The question of when to start intrathecal baclofen in this condition has not been addressed by published trials. While most often performed much later in the disease, a case can be made for implanting a pump relatively early to prevent the development of contractures, with a view to maintaining upright gait and retaining the option of supporting weak knee extensors and ankle dorsiflexors with orthoses [48].

Once ankle contractures have developed, and people can no longer stand with knees and hips straight while their heels are on the ground, heel wedging needs to be incorporated into shoes or AFOs to compensate. The lighter “off the shelf” AFOs, or FES of the peroneal nerve, are no longer appropriate in this situation as these only prevent passive plantarflexion during the swing phase of gait and are not designed to stabilise the foot and ankle during the stance phase of gait. The necessary custom moulded AFOs with compensatory heel wedging will often not fit in patients’ usual shoes; if larger trainers are impractical, expensive bespoke footwear may be required. Custom AFOs may also be designed to compensate for weak knee extensors using the ground reaction force to hold the lower leg in a more vertical position, but this is only possible if the knee still extends fully.

## Conclusion

The last decade has seen astonishing progress in the identification of HSP genes. Coupled to the introduction of high-throughput sequencing approaches, we are quickly moving towards the ideal situation where every HSP patient will have a defined molecular diagnosis if they choose to have it. This will give important immediate benefits to HSP families, including diagnostic certainty, prevention of unnecessary additional investigations and accurate risk prediction for clinically unaffected family members. In the future, as HSP therapies emerge, it may also be a pre-requisite for the personalised selection of appropriate treatment. In the meantime, it is important that supportive therapy, which can make a real difference to patients’ lives, is not neglected.
